# Acute Care QUAliTy in chronic Kidney disease (ACQUATIK): a prospective cohort study exploring outcomes of patients with chronic kidney disease

**DOI:** 10.1136/bmjopen-2014-006987

**Published:** 2015-05-02

**Authors:** Julia J Arnold, Manvir Hayer, Adnan Sharif, Irena Begaj, Mohammed Tabriez, David Bagnall, Daniel Ray, Ciaron Hoye, Masood Nazir, Mary Dutton, Lesley Fifer, Katie Kirkham, Don Sims, Jonathan N Townend, Paramjit S Gill, Indranil Dasgupta, Paul Cockwell, Charles J Ferro

**Affiliations:** 1 Department of Nephrology, Queen Elizabeth Hospital and University of Birmingham, Birmingham, UK; 2 Department of Informatics, Queen Elizabeth Hospital and University of Birmingham, Birmingham, UK; 3 Birmingham Crosscity Clinical Commissioning Group, Birmingham, UK; 4 Care of the Elderly Medicine, Queen Elizabeth Hospital and University of Birmingham, Birmingham, UK; 5 Department of Cardiology, Queen Elizabeth Hospital and University of Birmingham, Birmingham, UK; 6 Primary Care Clinical Sciences, University of Birmingham, Birmingham, UK; 7 Department of Nephrology, Heart of England NHS Foundation Trust and University of Birmingham, Birmingham, UK

**Keywords:** PRIMARY CARE

## Abstract

**Introduction:**

Chronic kidney disease (CKD) is common and carries a high risk of morbidity, including hospital admissions and readmissions and mortality. This is largely attributed to an increased risk of cardiovascular disease. Patients with CKD are less likely to receive evidence-based treatments for cardiovascular disease. However, these treatments are based on trials which generally exclude patients with CKD. It is therefore unclear whether this patient group derives the same benefits without an increased risk of adverse effects.

**Methods and analysis:**

The Acute Care QUAliTy in chronic Kidney disease (ACQUATIK) study is a prospective, observational, multicentre cohort study. Over 4000 patients will be recruited with an enrolment period of 2 years and a follow-up period of 2–4 years. Patients under follow-up by a renal team will be excluded. Data will be obtained from patient and hospital records during the index admission. Preadmission data will be extracted from general practice records based on the Quality and Outcomes Framework. Diagnosis, comorbidities and procedure data pertaining to the index and subsequent admissions will be extracted from the Hospital Episode Statistics database and long-term mortality data will be tracked using the Office of National Statistics. This information will allow us to examine a complete patient journey through primary and secondary care, providing unequalled levels of information on treatment and outcomes of patients with CKD. The combined data set will be used to compare outcomes and treatments among patients with CKD versus patients without CKD. The primary end point is hospital readmission rates. The relationship between age, sex, ethnicity, socioeconomic status and concurrent comorbidities will be analysed to determine their influence on outcomes and treatments.

**Ethics and dissemination:**

The ACQUATIK study has been approved by the NRES Committee West Midlands—South Birmingham—Reference 13/WM/0317. The results from ACQUATIK will be submitted for publication in peer-reviewed journals and presented at primary and secondary care conferences.

**Trial registration number:**

ISRCTN37237454.

**Strengths and limitations of the study**

- Novel research methodology facilitating patient recruitment with minimal inconvenience to patients and lower research costs.
- Large amounts of data available for analysis.
- Multicentre study recruiting from a large diverse population increasing the generalisability of the results.
- The observational nature of this study means that causality cannot be attributed.
- The use of routinely collected patient data, rather than data specifically collected as part of a research study may result in more missing information than would normally be expected.
- Patients who move away from England will be lost to follow-up.

## Introduction

### Chronic kidney disease

Current definitions of chronic kidney disease (CKD) use surrogates of kidney damage (comprising at least one of: proteinuria, abnormal urinary sediment, histological or structural abnormality or history of a renal transplant) and/or a glomerular filtration rate less than 60 mL/min/1.73 m^2^ present on at least two occasions for 3 months or more.^1^ ^2^ The prevalence of early CKD is increasing and now affects more than 13% of the population in the developed world.^3^ This increase has been largely driven by the increasing prevalence of the most common risk factors for CKD, including diabetes, hypertension and older age.^3^ Among adults aged more than 70 years, the prevalence of CKD has been estimated to be more than 40% in the USA and UK.^3^ For an important minority, CKD can progress to end-stage renal disease (ESRD) requiring renal replacement therapy (RRT) in the form of either dialysis treatment or kidney transplantation.^1^ The cost of CKD to the National Health Service (NHS) in England in 2009–2010 has been estimated at £1.45 billion^4^; indirect costs may be far higher.^5^ The shift in focus to active early detection and management of CKD, and other chronic conditions, has become an increasing priority for health services. Indeed, most patients with CKD are now managed in the primary care setting with little or no secondary care input.^6^ However, US data demonstrates that patients with CKD are more likely to be readmitted after a hospital admission than patients without CKD,^7^ with the associated increase in costs and comorbidity.

### Cardiovascular risk and CKD

A recent large meta-analysis has confirmed that for all adults, including those aged 75 years or older, absolute incidence rates of ESRD are substantially lower than absolute mortality rates.^8^ The major cause of this increased death rate has repeatedly been found to be cardiovascular disease.^8^ Over a 1-year period (2009–2010), approximately 7000 excess myocardial infarctions and 12 000 excess strokes occurred in patients with CKD in England compared to a non-CKD age and gender-matched population.^4^ The cost to the NHS of these extra events has been estimated at £178 million.^4^ This does not take into account other cardiovascular events such as sudden cardiac death or admissions for heart failure, nor the costs to individual patients, carers or society as a whole.

Outcomes for patients with CKD who suffer a myocardial infarction or stroke are consistently reported as worse than for patients without CKD.^9^ Studies in North America and Europe have found that patients with CKD are less likely to be treated with measures to prevent cardiovascular disease, are less likely to receive evidence-based treatments for a cardiovascular event when admitted to hospital and are less likely to be discharged with treatments aimed to prevent a further cardiovascular event.^10^ ^11^ There is emerging evidence that this is also the case in the UK.^12^

### Cardiovascular treatments in CKD

The low use of evidence-based treatments in patients with CKD might partly account for the worse outcomes in patients with CKD who sustain a cardiovascular event.^13^ However, most cardiovascular treatments are based on results from studies that have largely excluded patients with moderate to severe CKD, advanced age or multiple comorbidities.^14^ Furthermore, those trials that did include elderly and patients with CKD had high rates of patient withdrawal and early discontinuation of therapy.^15^ To complicate matters further, there is increasing evidence that patients with CKD may not benefit as much as patients without CKD from treatments for cardiovascular disease and also have an increased risk of suffering harm as a consequence.^16^ One example is the paradox of both an increased thrombotic tendency and an increased bleeding risk in patients with CKD and comorbidity of atrial fibrillation (AF).^17^ Another example was identified in a recent meta-analysis that suggested that the benefits of antiplatelet therapy in patients with CKD after an acute coronary syndrome are unresolved and are potentially overshadowed by an increased bleeding risk.^18^ Statins reduce atherosclerotic events in patients with CKD to a similar degree to the general population but reduce cardiovascular and total mortality by a substantially smaller amount.^19^

Thus, applying treatment strategies that are proven effective for the primary and secondary prevention of cardiovascular disease in patients without CKD to patients with CKD is challenging because of the lack of evidence of efficacy and the potential to do harm.^20^ Clinicians increasingly recognise that some treatments proven to be effective among middle-aged adults with normal kidney function may have different risks and benefits among older patients with CKD and multiple comorbidities.^16^

Explanatory trials often select narrow ranges of patients based on age, sex, comorbidity and concomitant treatments and then monitor patients carefully. In contrast, patients in routine clinical practice are more diverse, with varying disease histories and comedications with different levels of compliance and adherence. In fact, most explanatory studies exclude ‘average’ patients who will end up receiving the intervention tested in the trial.^21^ Furthermore, randomised controlled trials (RCTs) are not necessarily the best way of tackling certain research questions, such as what is the cardiovascular risk associated with CKD. Rigorous observational studies might be better suited for these purposes and in many situations produce comparable conclusions to RCTs.^22^ This type of study includes participants with a broader variety of disease severity and more comorbidities than patients normally recruited into RCTs.^23^

### Use of electronic health records in clinical research

Electronic information systems in healthcare are evolving and are increasingly used in routine clinical care, with primary care at the forefront in the UK. The richness and completeness of data held in computerised patient record systems has been increasing with time as more information is being shared electronically between different parts of the healthcare system and as paper-based patient records are being replaced by electronic patient records. For example, laboratory test results are increasingly being communicated electronically and loaded automatically into the electronic patient record. Use of these computerised health records can facilitate the conduct of trials as patients can be preidentified and followed up using routinely collected clinical data. The potential for using electronic health records to facilitate trials was highlighted over 10 years ago^24^ although at that time there were still substantial deficiencies in the routinely collected data, most of which have now largely been overcome.

The Acute Care QUAliTy in chronic Kidney disease (ACQUATIK) study provides a platform for linkage of: non-identifiable patient data from primary care derived from the Quality Outcomes Framework (QOF);^25^ electronically held records in secondary care and long-term outcomes from the Hospital Episode Statistics (HES) database in secondary care; and the Office of National Statistics (ONS). This information will allow the study of a complete patient journey through primary and secondary care, providing unequalled levels of information on treatment and outcomes of patients with CKD. Defining how CKD, older age, ethnicity and concurrent comorbidities modify the effectiveness and safety of today's common therapies will facilitate improvements in care for this group of patients.

Other studies have recently used existing electronic health record research databases to conduct clinical trials.^21^ A feasibility trial is currently ongoing using the USA Department of Veterans Affairs computerised patient record system.^26^ ACQUATIK has several potential advantages over these types of studies; in particular, the methods and procedures being developed are potentially exportable to most hospitals and certainly all primary care practices throughout England, allowing for every patient with any medical condition in the country to be eligible for participation in a research study. The unique methodology employed in ACQUATIK could therefore provide a platform for future interventional studies at the interface between primary and secondary care in the UK, allowing the seamless acquisition of large volumes of data at low cost.

## Methods and analysis

### Study design

The ACQUATIK study is a prospective, multicentre observational study of patients in the UK with and without CKD. Two main centres in Birmingham, University Hospitals Birmingham NHS Foundation Trust (UHBFT) and Heart of England NHS Foundation Trust (HEFT) are undertaking this study. Each centre has a high number of admissions and serves a large diverse population.

### Inclusion criteria

- Participant is an inpatient at either UHBFT or HEFT.
- Participant is willing and able to give informed consent to participate in the study.
- Men or women, aged 18 years or more.
- Patient is registered with a general practitioner (GP) in the two main Clinical Commissioning Groups (CCGs—clinically led groups that include all GPs in their geographical area) served by UHBFT and HEFT (Birmingham South Central CCG and Birmingham Crosscity CCG). Clinical Commissioning Groups were set up in April 2013 arising as part of the reforms set out in the Health and Social Care Act 2012 and replaced primary care trusts.^27^

### Exclusion criteria

To enhance inclusivity, there are only a few exclusion criteria:

- Unable to give informed consent.
- On dialysis or with a working kidney transplant.
- Already under long-term follow-up by a renal team in secondary care.
- Attended a renal clinic in secondary care in the preceding 12 months.
- Patients whom clinical staff responsible for their care feel are inappropriate for enrolment into the study.

### Study process

**Figure 1:**
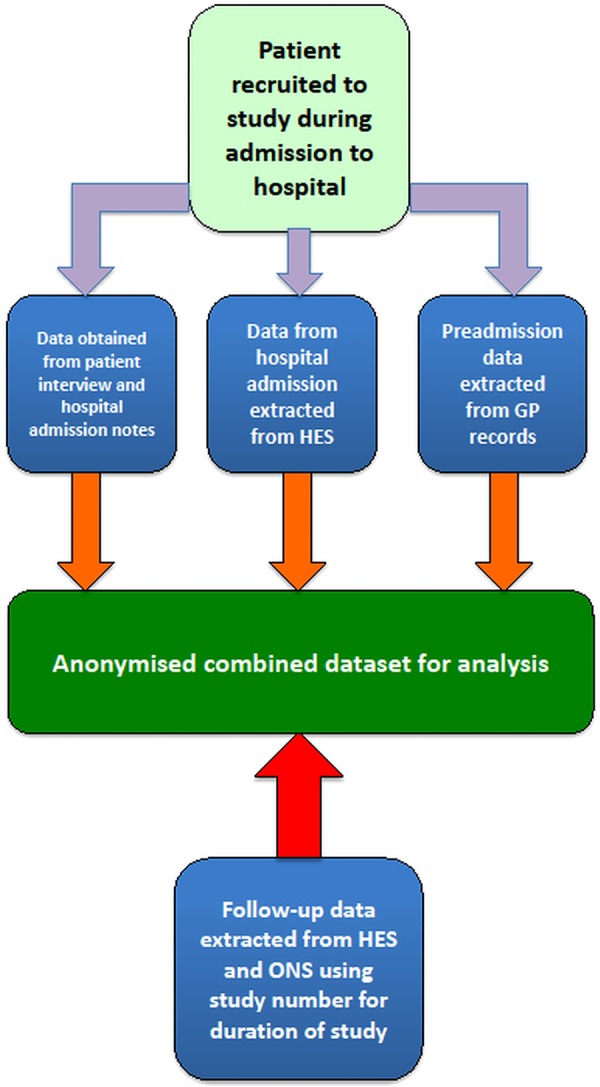
ACQUATIK study design.

The study process is laid out in figure 1.

1. Hospital electronic records are automatically searched for current inpatients who fulfil the study entry criteria. Patients who are under local renal follow-up or have attended a renal clinic in the preceding 12 months or who are not registered with a GP in the two CCGs involved are excluded at this stage.
2. Clinical staff responsible for the care of identified patients are approached to ensure they are appropriate for the study.
3. The research team approaches suitable patients. Where possible an introduction will be made by a member of the clinical team known to the patient. If there is any doubt about the participant's ability to give informed consent, they are excluded.
4. The researcher verbally explains the study and answers any questions the potential participant may have. The voluntary nature of participation and the ability to withdraw at any time is emphasised. As part of the consent process the patient is also asked to consent to the use of information held by the NHS and records maintained by the Health and Social Care Information Centre (HSCIC).^28^ The patient is given as much time as needed to think through their participation. After a suitable period, the patient is consented to participate in the study. Before consent, the researcher confirms with the participant that they have understood the patient information sheet.
5. During this visit, the patient and a member of the study team jointly fill in a questionnaire including demographic details, usual GP, lifestyle factors, kidney disease diagnosis and admission medication.
6. Data from biochemistry and haematology laboratory investigations are electronically collected including creatinine, estimated glomerular filtration rate, urea, albumin, haemoglobin, sodium, potassium, bicarbonate, phosphate, parathyroid hormone and cholesterol.
7. A letter is sent to the GP with a photocopy of the consent form. The GP surgery is asked to insert a Read code into the patient's electronic health record. This will be to register within the GP database that the patient has consented to be a part of the study and enables subsequent extraction of preadmission data based on the QOF and prescribed medication records. Once the study has ended or the patient withdraws from the study, a second Read code will be sent to the GP that will prevent any further data extractions. The UK Terminology Centre has provided these Read codes.
8. Preconstructed software already installed will automatically migrate the prespecified data to the Department of Informatics at UHBFT. All data will be transferred within the NHS N3 Network and held in a secure NHS hospital environment with strict restrictions on access and data reuse agreements.

### Data sources to be used

In addition to information obtained from the patient interview and electronic health records used during routine care, the following data sets will be accessed:

### Quality and Outcomes Framework (QOF)

This is a system for the performance management and payment of GPs in the NHS. It was introduced as part of the new general medical services contract in April 2004.^29^ Multiple clinical conditions are featured (see table 1) and it is a requirement for GPs to keep a database of all patients diagnosed. One example is CKD, where GPs are required to record all patients with an estimated glomerular filtration rate of less than 60 mL/min/1.73 m^2^ (stage 3 CKD or worse) on a practice register. This CKD record contains details of most patients labelled with CKD in the UK.^30^

**Table 1:** Quality and Outcomes Framework (QOF) chronic disease domains

QOF domain	Point for analysis
Chronic kidney disease (CKD)	Is the patient on the CKD register?
	If so, was the last BP reading (in the preceding 12 months) 140/85 mm Hg or less?
	If so, what was the last ACR (or PCR) in the preceding 12 months?
Atrial fibrillation (AF)	Is the patient on the AF register?
	If so, what is their CHADS2 score?
	Is the patient treated with anticoagulation drug therapy or antiplatelet therapy?
Secondary prevention of coronary heart disease (CHD)	Is the patient on the CHD register?
	If so, what was the latest BP (within 12 months)?
	If so, what was the last measured cholesterol (within 12 months)?
	If so, has the patient received influenza vaccination in the previous 12 months?
	If so, is the patient on aspirin, alternative antiplatelet agent or oral anticoagulant?
Heart failure (HF)	Is the patient on the HF register?
	If so, is the patient currently treated with an ACE-I or ARB?
	If on ACE-I or ARB are they also treated with a β-blocker?
Hypertension (HYP)	Is the patient on the hypertension register?
	If so, has the BP measured in the preceding 9 months been less than 150/90 mm Hg?
Peripheral arterial disease (PAD)	Is the patient on the PAD register?
	If so, has the BP in the preceding 12 months been 150/90 mm Hg or less?
	If so, has the total cholesterol measured in the preceding 12 months been 5 mmol/L or less?
	If so, is the patient taking aspirin or an alternative antiplatelet agent?
Stroke and transient ischaemic attack (STIA)	Is the patient on the STIA register?
	If so, is the last BP reading measured in the preceding 12 months 150/90 mm Hg or less?
	If so, what was the last measured cholesterol (within 12 months)?
	If so, was the last measured cholesterol (within 12 months) less than 5 mmol/L?
	If so, has the patient received influenza vaccination in the previous 12 months?
	If so, is the patient on an antiplatelet agent or oral anticoagulation?
Diabetes mellitus (DM)	Is the patient on the DM register?
	If so, is the last BP reading measured in the preceding 12 months 150/90 mm Hg or less?
	If so, is the last BP reading measured in the preceding 12 months 140/80 mm Hg or less?
	If so, was the last measured cholesterol (within 12 months) less than 5 mmol/L?
	If so, was the IFCC-HbA1c less than 59 mmol/mol in the preceding 12 months?
	If so, was the IFCC-HbA1c less than 64 mmol/mol in the preceding 12 months?
	If so, was the IFCC-HbA1c less than 75 mmol/mol in the preceding 12 months?
	If so, has the patient received influenza vaccination in the previous 12 months?
Hypothyroidism (THY)	Is the patient on the THY register?
	If so, have they had a thyroid function test recorded in the preceding 12 months?
Asthma (AST)	Is the patient on the AST register?
Chronic obstructive pulmonary disease (COPD)	Is the patient on the COPD register?
	If so, what was the last measured (within 12 months) FEV_1_?
	If so, has the patient received the influenza vaccination in the previous 12 months?

ACR, urinary albumin: creatinine ratio; ARB, angiotensin receptor blocker; BP, blood pressure; CHADS2, C, congestive cardiac failure, H, hypertension (treated or untreated (BP ≥140/90 mm Hg), A, age ≥75 years, D, diabetes mellitus, 2, previous stroke, TIA or thromboembolism; FEV_1_, forced expiratory volume in 1 second; IFCC-HbA1c, International Federation of Clinical Chemistry glycated haemoglobin.

### Hospital Episode Statistics

Hospital Episodes Statistics (HES) is an administrative data set that collates information on all patients admitted to all NHS hospitals in England. Each admission contains a primary diagnosis and up to 19 secondary diagnoses (recorded using the standard system of International Classification of Diseases, 10th edition), and up to 24 procedure fields. In addition to diagnostic and procedural codes, the HES database^31^ also records the Charlson comorbidity index. Derived from the secondary diagnoses codes, this is a marker of comorbidity and was originally formulated to predict mortality.^32^ The Carstairs index of deprivation is derived from the patient's postcode.^33^ As well as clinical information, each HES record will also contain routine information about the patient (eg, age, sex, ethnicity and postcode) and the episode of care (eg, hospital name, emergency admission, date of admission and discharge). All hospitals in England are required to submit data to HES.

### Office of National Statistics

All deaths in England must be notified by law and recorded by the Office for National Statistics (ONS). Deaths during the study period, including date and cause of death, will be derived through linkage between HES records and death registry information via the ONS. This improves mortality capture by including deaths outside of hospitals.

Information from these three databases has been collated by systems developed at one hospital (UHBFT) and one CCG and these are being tested at a second hospital (HEFT) and CCG. ACQUATIK aims to develop systems that are potentially exportable to other hospitals and CCGs in England and use these to conduct large multicentre interventional trials covering wider geographical areas in the future.

## Outcome measures

### Primary outcome

Hospital readmission rates in patients with and without CKD.

### Secondary outcomes

1. Hospital readmission rates for any cardiovascular event (including acute myocardial infarction, angina, coronary artery bypass graft surgery, percutaneous coronary intervention, peripheral arterial disease, revascularisation or stroke) in patients with and without CKD.
2. All-cause and cardiovascular mortality in patients with and without CKD.
3. Treatment received before, during and after admission to hospital with a cardiovascular event by patients with and without CKD.
4. The risk: benefit ratio for primary and secondary prevention of cardiovascular disease in patients with and without CKD.
5. Attainment of standards in chronic disease management in patients with and without CKD.
6. Rates, severity and risk factors for acute kidney injury in patients with and without CKD.
7. The proportion of patients with biochemical evidence of CKD not classified so by the GP.

All primary and secondary outcomes will be analysed separately to investigate the influence of age, sex, ethnicity, socioeconomic status, deprivation score and concurrent comorbidities (including diabetes mellitus) on outcomes, treatment and classifications. Determination of socioeconomic status will be based on the Index of Multiple Deprivation model calculated at the local level.^34^

In the ACQUATIK study patients with renal disease under secondary care nephrology follow-up have been excluded. Therefore, the majority of diagnoses coded within HES and primary care databases will include common systemic conditions such as hypertension and diabetes mellitus as the aetiology of CKD, rather than primary renal glomerulonephritides which would invariably be under secondary care follow-up in the UK. Conditions such as diabetes mellitus will also be present in the non-CKD group and will form part of the prespecified analysis of comorbidities during data analysis.

Total hospital readmission rate was selected as the primary end point rather than readmission for cardiovascular events for the following reasons. First, ACQUATIK was powered on total readmission rate of patients with and without CKD as this is the available data. Second, hospital readmission rate is more a robust measure than an admission for a cardiovascular event—which would be regarded as a secondary classification and based on a single primary admission code—especially if a patient presents with more than one condition simultaneously. For example, an admission with a hip-fracture would be coded as such and may not necessarily reflect an underlying cardiovascular cause such as a cardiac arrhythmia or iatrogenic hypotensive episode. Third, ACQUATIK seeks to ascertain readmissions as a result of complications of treatments for cardiovascular disease, for example, gastrointestinal haemorrhage following antiplatelet therapy for acute myocardial infarction. These two admissions (hip-fracture after a hypotensive episode and gastrointestinal bleed with aspirin), although highly relevant to our study, would not have formed part of our primary end point. Finally, hospital readmission for any cause is seen as a marker of quality of care.^35^ This is the main aim of the ACQUATIK study.

### Follow-up period and study end

The patient enrolment period is 2 years. Patients will be followed until death, withdrawal from the study, or for a maximum of 4 years, whatever comes first. For patients who died during follow-up, information about the causes of death will be collected. For patients who wish to stop participation in the study, the reason for withdrawal will be registered. The end of the study will be defined as the end of long-term follow-up 2 years after the last patient was recruited into the study. The data generated from this study will be held in a secure, anonymised database for 2 years after the study ends. The database will then be archived for a total of 10 years as per sponsor guidelines after the study ends to protect the integrity and auditability of the research. However, given the likelihood that this data will be of great value for future research the Chief Investigator will be applying for permission to maintain the database, under the relevant conditions, to the appropriate regulatory bodies.

### Number of patients

A minimum of 4000 patients will be recruited into the study. The sample size calculations were based on the following information. Approximately 40% of patients admitted to hospital will have evidence of CKD (data from 4 years of emergency admissions to Queen Elizabeth Hospital). This is consistent with published international data.^36^ From US Medicare data,^7^ patients without CKD aged 66 and over have a readmission rate of 82 per 1000-patient years whereas patients with CKD have a readmission rate of 116 per 1000-patient years. From US Marketscan data^7^ patients without CKD aged 50 to 64 have a readmission rate of 56 per 1000-patient years whereas patients with CKD have a readmission rate of 72 per 1000-patient years. Therefore, in a best case scenario, patients admitted to hospital in Birmingham have annual readmission rates similar to US Medicare data. To detect a difference of 34 (116–82) readmissions per 1000 patient-years with 92% power would require a sample size of 2000 with an average of 2 years follow-up. In a worst case scenario, patients admitted to hospital in Birmingham have annual readmission rates similar to US Marketscan data. To detect a difference of 16 (72–56) readmissions per 1000 patient-years with 80% power would require a sample size of 4000 with an average of 2 years follow-up.

## Analyses

Normally distributed continuous data will be presented as mean±SD with comparisons made using Student's t test or one-way analysis of variance. Non-normally distributed data will be presented as median (IQR) and analysed using the Kruskal-Wallis test. Categorical data will be presented as count (percentage) and analysed using the χ^2^ test. In multivariable analyses, variables found to be significantly associated with the outcome under investigation in the univariable analyses will be used. Logistic regression analysis will be used to assess the relationship between outcomes and parameters under investigation and the results expressed as an OR with 95% CIs. Time-to-event data analysis will be performed using the Cox proportional hazards model and the results expressed as HR with 95% CIs.

Standard methods will be used to handle missing data after being assessed for extent and as to whether this is due to random missingness and/or censoring.^37^ ^38^

## Ethics and dissemination

### Ethics

The Chief Investigator will guarantee that the study is conducted in accordance with the Declaration of Helsinki and will ensure that all staff involved in the study are appropriately trained in Good Clinical Practice (GCP). All staff will adhere to Caldicott principles, GCP and the Data Protection Act, 1998 ensuring patient confidentiality. If needed, the Chief Investigator will submit and gain approval for all substantial amendments to the original approved documents. All participants are identified on study documents by use of a unique study number that cannot be used to identify individual participants. All study documents, both electronic and paper, containing participant information are held securely and are only accessible to study staff and authorised personnel. All essential data transfer happen within the secure NHS N3 Network.

All patient identifiable data will be held separately from the information collected on the patient and will be used only if a member of the research team needs to recontact the patient for any potential follow-up study (specific consent will be obtained from participants for this purpose). Patient identity will not be revealed in publications arising from the study.

The study is registered on the ISRCTN (International Standard Randomised Control Trial Number) database (ISRCTN37237454).

### Dissemination

In accordance with the Research Governance Framework for Health and Social Care and GCP, results will be appropriately published and publicised. The investigators will be involved in reviewing all publications arising from the study. The authors of such publications will acknowledge that the study is funded through a National Institute for Health Research (NIHR) Fellowship Grant awarded to the Chief Investigator as well as a strategic funding award from the West Midlands (formerly Birmingham and Black Country) Comprehensive Local Research Network.

The results from this study will be important for both primary and secondary care communities. The findings will be presented at both primary and secondary care academic meetings. It is anticipated that this study will produce manuscripts suitable for submission to relevant peer-reviewed journals. It is our intention, if at all possible, to publish in open access journals to encourage widespread diffusion of our findings.
